# The effect of natural surfactants on the development of postoperative intraabdominal adhesion

**DOI:** 10.55730/1300-0144.5676

**Published:** 2023-04-11

**Authors:** Ahmet Gökhan GÜLER, Ali Erdal KARAKAYA, Ahmet Burak DOĞAN, Abdülkadir Yasir BAHAR, Sadık YURTTUTAN

**Affiliations:** 1 Department of Pediatric Surgery, Faculty of Medicine, Sütçü İmam University, Kahramanmaraş, Turkiye; 2 Department of Pediatric Surgery, Faculty of Medicine, Erciyes University, Kayseri, Turkiye; 3 Department of Pathology, Faculty of Medicine, Sütçü İmam University, Kahramanmaraş Turkiye; 4 Department of Neonatology, Faculty of Medicine, Sütçü İmam University, Kahramanmaraş Turkiye

**Keywords:** Postoperative adhesion, surface-active phospholipids, pulmonary surfactant, beractant, poractant, calfactant

## Abstract

**Background/aim:**

The development of postoperative adhesion after abdominal surgery is sometimes a severe problem. Our study investigates the effectiveness of exogenous surfactant application in preventing adhesion development in the experimental adhesion model.

**Materials and methods:**

This randomized-controlled interventional study was carried out in the animal laboratory of Kahramanmaraş Sütçü İmam University between March 1 and March 31, 2020. An experimental intra-abdominal adhesion model was established in 24 adult female rats by cecal abrasion. Rats were randomly divided into four groups. Groups I, II, and III were taken intraperitoneally as beractant, poractant, and calfactant applied groups, respectively. Group IV was the control group. Relaparotomy was performed in all groups on the 15th postoperative day, and intra-abdominal adhesions were scored macroscopically according to the Canbaz scoring system. In addition, the cecal regions were evaluated microscopically and scored according to the Zühlke microscopic classification system. The scores of the groups were compared statistically.

**Results:**

The Zühlke adhesion development score was significantly lower in the exogenous surfactant applied groups. In addition, when the surfactant-applied groups were compared among themselves, it was seen that the adhesion score in the beractant group was significantly better than the other surfactant types (p < 0.01).

**Conclusion:**

Our study results showed that prophylactic intraperitoneal surfactant application significantly reduced postoperative adhesion development, particularly beractant.

## 1. Introduction

Despite advances in surgical techniques, adhesion development after abdominal surgery remains a problem [1–4]. Peritoneal damage during surgery, damaged serosal surfaces, tissue ischemia, the presence of foreign materials in the environment, increased vascular permeability on the background, fibroblastic activity with underlying decreased fibrinolytic activity with growth factors, and inflammatory cytokines can cause intra-abdominal adhesions [5]. The most common clinical problems related to postoperative intra-abdominal adhesions in all age groups are abdominal pain, nutritional intolerance, intestinal obstruction findings, female infertility, and the need for relaparotomy [6–8].

Many agents, such as phospholipase inhibitors and dextran, have been investigated as inhibitors of postoperative intra-abdominal adhesion formation [1,2,9]. Surface-active phospholipids, which have excellent release and lubricant properties, were identified as surfactant-like substances in the peritoneal dialysis effluent [2,10]. Phosphatidylcholine (PC) is the main constituent of the surface-active material coating peritoneal mesothelium. It may prevent postoperative adhesion formation by producing a lubricant film on mesothelial defects [11]. The surface-active phospholipid layer acts as a boundary lubricant and release (antiadhesive) agent that maintains the mechanical integrity of the epithelial surface, thereby preventing the formation of adhesion bridges between two solid structures [12,13].

Pulmonary surfactant contains a multicomponent complex of several phospholipids, neutral lipids, and specific proteins. The main functions of pulmonary surfactant are reducing the collapsing force in the alveolus, conferring mechanical stability to the alveoli, and maintaining the alveolar surface relatively free of liquid. Previous studies have shown the inhibitory effects of exogenous surfactants in experimental adhesion models [2,3].

There are three surfactants of exogenous natural origin: 1-beractant, 2-poractant, and 3-calfactant. There is no postoperative adhesion study in the literature evaluating all of them comparatively.

In this study, we aimed to evaluate the effects of all-natural exogenous surfactants used in the clinic for a long time on developing postoperative adhesion, revealing the possible difference between them.

## 2. Materials and methods study design

This randomized-controlled interventional study was carried out after the approval of the Experimental Animals Ethics Committee of our institute (date: 08/05/2019-decision no: 02) in the animal laboratory of Kahramanmaraş Sütçü İmam University between March 1 and March 31, 2020. As in the similar study of Yılmaz et al. adult female Wistar-Albino rats, each weighing between 240–260 g, were used in the study [2]. Rats were maintained in cages in an environment with controlled temperature (21 °C ± 2 °C) and humidity (55.5%) with a 12-h light/dark cycle. They were fed with commercial food and water ad libitum. A heating lamp was used to preserve the body temperature at 37 °C. All animals received humane care in compliance with the Principles of Laboratory Animal Care by the National Society for Medical Research and Guide for the Care and Use of Laboratory Animals prepared by the Institute of Laboratory Animal Resources [14]

A total of 24 rats, including four groups and six randomly selected in each group, were used in the study.

### 2.1. Surgical technique

**Figure 1:**
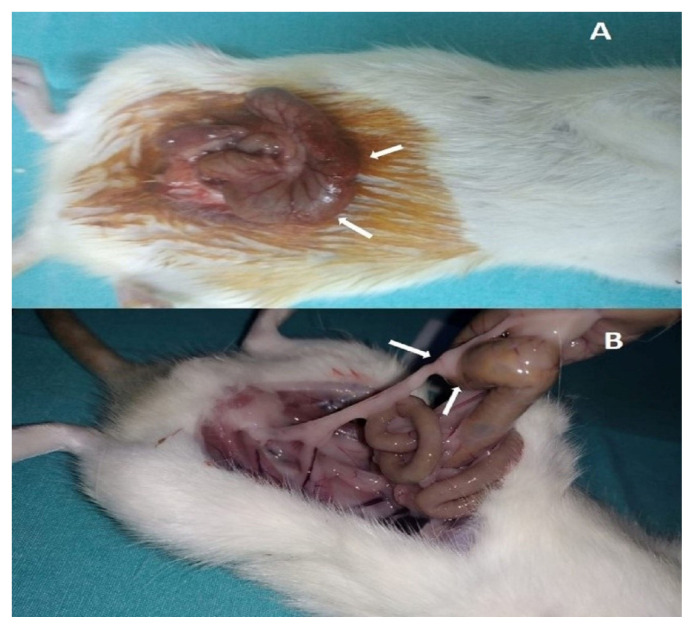
Cecal abrasion technique (A), Postoperative adhesion (B)

An intra-abdominal adhesion model was created as described by Hemadeh et al. [15]. The surgeries were performed under intramuscular administration of 50 mg/kg ketamine hydrochloride (Ketalar; Eczacıbaşı, İstanbul, Türkiye) and 5 mg/kg xylazine hydrochloride (Alfazyne; Ege Vet, İzmir, Türkiye). The same surgeon performed all operations to ensure technical uniformity (A.G.G). After sterile site cleaning, the terminal ileum and cecum were removed after a 3.0 cm long abdominal midline incision. The anterior surface of the cecum was abraded with a dry gauze pad until the serosal shine disappeared, and punctate hemorrhages were seen (Figure-1A). A drop of absolute alcohol was applied to promote adhesion formation. Then, the cecum and ileum were placed into the abdomen after using the required surfactant types for each group and 0.09% NaCl for the control group. The abdominal incision was closed in 2 layers with continuous 3–0 silk sutures.

### 2.2. Surfactan application

The intraperitoneal surfactant doses were taken from a similar study in the literature [2]. In the study groups, 100 mg/kg of surfactant was diluted with 0.9% NaCl to 2 mL. Some of this mixture was dripped onto the wound site; the rest was applied to the intestinal surface by spray intraperitoneally.

Group 1 (beractant-applied group): After surgery of intra-abdominal adhesion, 100 mg/kg beractant was administered intraperitoneally by diluting with 0.9% NaCl. (Survanta; Abbott Laboratories, Chicago, IL).

Group 2 (poractant-applied group): After surgery of intra-abdominal adhesion, 100 mg/kg poractant was administered intraperitoneally by diluting with 0.9% NaCl. (Curosurf; Chiesi Farmaceutici, Parma, Italy).

Group 3 (calfactant-applied group): After surgery of intra-abdominal adhesion, 100 mg/kg calfactant was administered intraperitoneally by diluting with 0.9% NaCl. (Infrasurf; Farma Tek İlaç Sanayi, İstanbul, Türkiye).

Group 4 (control group): After surgery of intra-abdominal adhesion, 2 ml of 0.9% NaCl was administered intraperitoneally.

After 15 days, all animals were humanely sacrificed by cervical dislocation under anesthesia. The abdominal cavity was examined after making an inverted U-shaped incision to visualize the adhesive bands in the entire abdomen easily (Figure-1B). Adhesions were evaluated and scored by one of the authors blinded to the group assignment. Macroscopic scoring was performed by taking the total score according to the modified scoring system described by Canbaz et al. [16]. Each cecum was removed asen bloc and subjected to standard and immunohistochemical histological evaluation. Histopathological scoring was done according to the scoring system described by Zühlke et al. [17]. Exclusion criteria were intra-abdominal adhesions at the first surgery and postoperative problems such as wound infection, peritonitis, perforation, or death before the 15th postoperative day.

### 2.3. Histopathological examination

Tissue samples were taken from the cecum of all animals for light microscopy analysis. To avoid mucosal damage, the intestinal lumen was carefully cannulated and gently rinsed with normal saline solution before sampling. Cecum samples were fixed in a 10% neutral buffered formalin solution for 24 h. After dehydration, samples were fixed in paraffin with increasing concentrations of ethanol and xylene. 3.5 μm thick transverse sections were prepared from paraffin-embedded tissues with Leica RM 2125 RT (Bensheim, Germany). Randomly selected slices were stained with hematoxylin, eosin (H&E), and Masson’s trichrome (MT). Histopathological studies were performed by a single pathologist (A.Y.B) blinded to the group allocation and examined with a Nikon Eclipse Ni (Japan) light microscope. According to Zühlke scoring system used in histopathological evaluation; grade 0: no adhesion, grade 1:a loose connective tissue with fibrin, rich in inflammatory cells and fine reticulin fibers, grade 2: a connective tissue rich in fibroblasts, in addition to capillaries and inflammatory cells, few early collagen fibers, grade 3: a vascular-rich connective tissue with increased elastic and collagen fibers, decreased amount of inflammatory cells, grade 4: a granulation tissue poor in inflammatory cells, consisting of thick collagen fibers that are difficult to distinguish from serosal connective tissue [17]. Histological images were photographed with a Nikon Ds-Fi3 camera

### 2.4. Statistical analysis

Statistical analysis was performed using the SPSS for Windows version 20.0 software (IBM Corp., Armonk, NY, USA). Nominal variables between groups were analyzed using Kruskal-Wallis and Jonckheere-Terpstra tests. Comparison of adhesion scores between 2 groups was examined using the Mann-Whitney U test, where the data were nonnormal. A pvalue of less than 0.05 indicated statistical significance.

## 3. Results

All rats completed the study. The two scoring data of all groups included in the study are shown in Table. There was a significant difference between the groups. There is a significant difference between the groups with the Jonckheere-Terpstra test (p < 0.042), beractant was found to be significantly more effective than poractant and calfactant in pairwise comparisons with Mann-Whitney U test based on Zühlke scores (p < 0.043). No significant difference between groups according to Canbaz scores.

**Figure 2:**
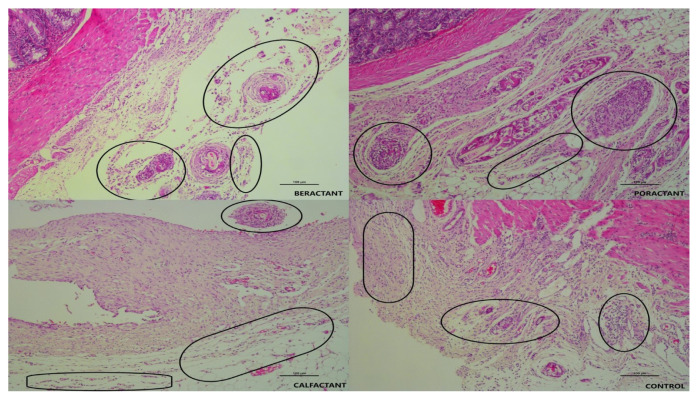
On microscopic evaluation; inflammatory cell accumulation (in the circle) in the periserosal tissue was evaluated, and small polymorphonuclear cell clusters and giant cell reactions were observed in the adipose tissue in the beractant group. In the poractant and calfactant groups, there was a slight increase in the number of inflammatory cells compared to the beractant group, while crowded inflammatory cell clusters were observed in the fibrous tissue in the control group. (Hematoxylin and Eosine, 10x objective)

**Figure 3:**
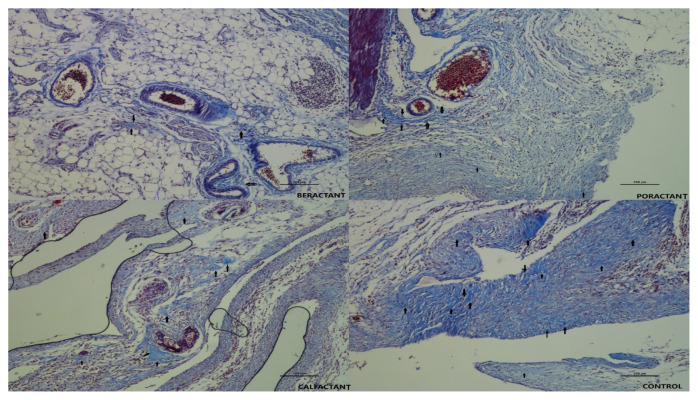
As the histopathological presentation of adhesion, collagen deposition was evaluated with Masson’s trichrome stain, periserosal adipose tissue was preserved in the beractant group and dark blue collagen deposition (marked with black arrows) was only in the form of tiny fine fibers. In the poractant and calfactant groups, thicker fibers were seen. With a significant decrease in adipose tissue in the control group, also there are wide and dark collagen fibers seen. (Masson’s Trichrome Stain, 10x objective)

The development of an organized fibrotic process associated with advanced inflammation was reduced in all groups treated with surfactant preparation in our study. Microscopically, inflammation was limited to the peri-serosal region in all surfactant groups, and muscularis propria, submucosa, lamina propria, and mucosal layers were regular. Severe collagen deposition was not present. In the control group, inflammatory granulation tissue formation corresponded to the early and middle stages of periserosal inflammation, wound healing, and collagen deposition. Thick collagen-rich cell-poor granulation tissue consistent with Grade 4 was not observed. Macrophage-dominated chronic inflammatory cell accumulation and focal foreign body reaction were more prominent in the beractant group compared to the other groups. At the same time, fibroblastic activation, neovascularization, and collagen production were higher in the calfactant, poractant, and control groups, and Zühlke’s score averages were close (Figure 2, hematoxylin-eosin staining). Irregular collagen bundles, containing many fibroblasts, extending from the serosa to the subserosal adipose tissue, consistent with Grade 2 and Grade 3 collagen bundles with thicker and fewer fibroblasts were frequently seen in the calfactant, poractant, and control groups, but not in the beractant group (Figure 3). The mesothelial layer is intact in all samples.

## 4. Discussion

Our study used three natural surfactant molecules in the postoperative abdominal adhesion model. We found the rate of intraperitoneal adhesion to be lower in all groups administered intra-abdominal surfactant compared to the control group. When the groups’ intraperitoneal surfactants were evaluated, adhesion was significantly lower in the group, mainly using beractant, compared to other surfactant molecules (Table). This is the first study in the literature to assess the effects of all-natural surfactants used in neonatal units on postoperative adhesion development.

Alveolar surfactant is a vital molecule with its reducing surface tension, coating, and at the same time, immunoregulatory properties with its phospholipid, neutral lipid, and specific protein contents [18]. Natural pulmonary surfactants have been used successfully in neonatal respiratory distress syndrome for a long time. Its phospholipids have excellent coating, limiting, lubricant, and antistatic properties [19–21].

Postoperative adhesion and stricture are important complications that can develop after surgery in pediatric cases as in all age groups. Antiadhesive applications used in other age groups cannot be used in pediatric and, particularly, neonatal patients due to pathophysiological differences in abdominal surgical interventions [22]. However, a gold standard application that can also be used for postoperative adhesion development in pediatric age groups has not been defined [23]. The search for an ideal and risk-free antiadhesive agent to be preferred in this special and sensitive patient group continues.

Although postoperative adhesion is a problem encountered from time to time in all pediatric age groups, it is observed more frequently in the infantile patient group due to the nature of the diseases [24]. In neonatal cases, the majority of them are asymptomatic, and the adhesion rate of different degrees was determined as 79% [25]. While most of them can be followed with conservative treatment without secondary intervention, it sometimes contributes to the development of postlaparotomy small bowel obstruction in infants, particularly in the first two years [25,26]. The incidence of clinically significant postoperative adhesions in infants is around 10%. In addition, higher rates have been reported mainly in operated gastroschisis and NEC cases. If postoperative adhesion development requires intervention, the risk of adhesion development increases further in the following process [27]. In addition to the problems in the follow-up processes of the patients, the treatment processes of these patients can also cause substantial economic losses. During the development of postoperative adhesion, damaged serosal surfaces, increased vascular permeability, and collagen matrix production with fibroblasts occur with the underlying decreased fibrinolytic activity with growth factors and inflammatory cytokines [28].

Based on this physiopathology background, many molecules that may be effective in different age groups in the development of postoperative adhesion have been studied; barrier films, fibrinolytics, antiinflammatory agents, anticoagulants, antioxidants, and phospholipids [3]. Phospholipids have self-assembly, emulsifying, and wetting, originating from their amphiphilicity properties. They can close serosal defects and are unique with their lubricating properties. Phospholipids are synthesized from mesothelial cells [29]. In addition, it has been speculated that the peritoneal-surface coating surfactant is rich in PC, is a thin layer that coats the peritoneal surfaces by being absorbed by the mesothelium, and has chemical properties similar to pulmonary surfactant [30,31]. We think the postoperative antiadhesion properties of surfactant were more pronounced in the beractant group and provided better pathological results in all surfactant groups.

With the increased permeability on intraperitoneal serosal surfaces after surgery, cytokines, reactive immune cells, exudate, and fibrin play a role in the adhesion development process. Intraperitoneally administered surfactant dilutes these molecules and may reduce the organized inflammation development and associated adhesion development risk by negatively affecting cell migration. We speculated that the intraperitoneal surfactant preparation we applied in our study decreased the adhesion score by acting with this mechanism. This effect was highest in the beractant-treated group because this group has the highest surfactant volume compared to the other surfactant-treated groups. This effect was more pronounced in the beractant-treated group as shown in Table because this group has the highest surfactant volume compared to the other surfactant-treated groups.

The positive effects of antiinflammatory applications on the development of postoperative adhesion have also been studied. Studies in the literature show that surfactant preparations in different tissues can prevent exaggerated inflammation with the immuno-regulation process [32]. Although its effect on intestinal inflammation has not been defined concretely, the development of an organized fibrotic process associated with advanced inflammation was reduced in all groups treated with surfactant preparation in our study. This decrease was more pronounced in the beractant. There are also studies in the literature that enteral phospholipid supplementation reduces intestinal inflammation [33]. It can be speculated that this effect may be related to the phospholipids in the surfactant content.

The coagulation process is very determinant in the postoperative adhesion development process. Surfactant has been shown to prevent clot formation by negatively affecting the coagulation process in vitro studies [34]. However, fibrin formation is a determinant in the adhesion development process. In this process, fibronectin is quite active. It has been stated that applying surfactant can reduce fibronectin-fibrin binding [35]. Based on the macroscopic evaluation, fibrin development was decreased in all study groups given surfactant. We believe that intra-abdominal surfactant administration may have reduced fibrin development by affecting coagulation steps.

The lubricant effect of surfactant between tissues is important. The surface lubricity of the visceral organs in contact with each other is extremely important. Phospholipids have also been shown in the intra-pleural space and visceral-parietal surfaces [12]. Similarly, intestinal organ motility was stated to increase with intraperitoneal surfactant application. We believe that coating the visceral surfaces with intraperitoneal lubrication, preventing secondary visceral damage, and easier organ movements may have prevented the development of fibrin and organized fibrosis. In our study, beractant was more positive than the other surfactant groups. We believe that the volume of the dose used here is important. Beractant was given at 25 mg/mL, calfactant at 35 mg/mL, and poractant at 80 mg/mL. All molecules completed to 2 mL with 0.9% NaCl. Therefore, the volume of surfactant was the highest in the beractant group. We consider that this group’s physical lubricant and antithrombotic efficacy are more pronounced due to the more prominent serosal coating.

Yilmaz et al. previously applied intraperitoneal surfactant in a rat model [2]. Only beractant and poractant were compared in their studies. In that study, no significant difference was observed between the poractant and beractant applied groups. In our study, however, there was a significant difference in adhesion score in the beractant group compared to the poractant and calfactant groups (Table). This result can be attributed to the adequate surfactant volume in beractant.

The weak point of our study is the lack of fibrin, collagen, and severity of inflammation scoring system in the study. The severity of fibrin growth, collagen, and severity of inflammation are based on the macroscopic evaluation. Another weak point is the use of adult rats in the study. Although it is more appropriate to evaluate the intra-abdominal surfactant efficacy in newborn rats, especially in the infantile age group, applying the postoperative adhesion model to newborn rats is technically impossible.

We have shown in an experimental model that all pulmonary surfactants effectively prevent adhesions. Further animal and clinical studies are needed to corroborate these findings and determine the efficacy, safety, and most appropriate dosage of pulmonary surfactants in preventing postoperative adhesions.

## Conclusion

Our study showed that prophylactic intraperitoneal surfactant application significantly reduced postoperative adhesion development, particularly beractant. We believe that preventative intraperitoneal application of poractant and calfactant, foremost beractant, may be beneficial for developing postoperative adhesion, particularly in sensitive infantile patient groups with a high risk of adhesion. We believe clinical studies on this subject would yield more accurate results for the perioperative use of natural surfactants.

## Figures and Tables

**Table:** Canbaz and Zühlke scores of all study groups.

Zuhlke Microscopic Adhesion Score	Beractant	Poractant	Calfactant	Control
0	-	-	-	-
1	3	2	2	-
2	3	4	3	5
3	-	-	1	1
4	-	-	-	-
Mean ± SD	1.50 ± 0.54*	1.66 ± 0.51	1.83 ± 0.40	2.16 ± 0.40*
Canbaz Macroscopic Adhesion Score				
0	1	1	-	-
1	3	3	2	3
2	1	-	4	1
3	-	2	-	-
4	-		-	2
Mean ± SD	1.16 ± 0.75	1.83 ± 0.75	1.66 ± 0.81	2.16 ± 1.47

* p < 0.05
